# Oxytocin modulates meta-mood as a function of age and sex

**DOI:** 10.3389/fnagi.2015.00175

**Published:** 2015-09-10

**Authors:** Natalie C. Ebner, Marilyn Horta, Tian Lin, David Feifel, Håkan Fischer, Ronald A. Cohen

**Affiliations:** ^1^Department of Psychology, University of FloridaGainesville, FL, USA; ^2^Cognitive Aging and Memory Program, Clinical Translational Research Program (CAM-CTRP), Institute on Aging, Department of Aging and Geriatric Research, University of FloridaGainesville, FL, USA; ^3^Department of Psychiatry, University of CaliforniaSan Diego, CA, USA; ^4^Department of Psychology, Stockholm UniversityStockholm, Sweden

**Keywords:** oxytocin, aging, sex, emotion, meta-mood

## Abstract

Attending to and understanding one’s own feelings are components of meta-mood and constitute important socio-affective skills across the entire lifespan. Growing evidence suggests a modulatory role of the neuropeptide oxytocin on various socio-affective processes. Going beyond previous work that almost exclusively examined young men and perceptions of emotions in others, the current study investigated effects of intranasal oxytocin on meta-mood in young and older men and women. In a double-blind between-group design, participants were randomly assigned to self-administer either intranasal oxytocin or a placebo before responding to items from the Trait Meta-Mood Scale (TMMS) about attention to feelings and clarity of feelings. In contrast to older women, oxytocin relative to placebo increased attention to feelings in older men. Oxytocin relative to placebo enhanced meta-mood in young female participants but reduced it in older female participants. This pattern of findings supports an age- and sex-differential modulatory function of the neuropeptide oxytocin on meta-mood, possibly associated with neurobiological differences with age and sex.

## Introduction

There is growing evidence that the neuropeptide oxytocin functions within the social and affective systems of animals and humans as a key neuromodulator involved in the synchronization of body and brain in the coordination of a wide spectrum of behavioral, physiological, and psychological responses across a variety of life circumstances (Panksepp, 2009; Meyer-Lindenberg et al., 2011). In particular, oxytocin appears to promote social approach and reduce anxiety, possibly by increasing salience of socio-affective information and augmenting reward neural circuits (De Dreu, 2014). This neural model is supported by evidence that brain regions such as the amygdala and nucleus accumbens that are associated with socio-affective and reward processing are particularly sensitive to oxytocin (Gimpl and Fahrenholz, 2001; Balleine et al., 2007).

It has been proposed that oxytocin may primarily affect lower brain mechanisms and, via this route, also modulate higher-cognitive processes. That is, neural processes that generate basic emotional states (i.e., primary process of feeling) interact with higher neocortical substrates that integrate cognitive and cultural contributions to emotions in humans (i.e., secondary process of learning and thinking as well as tertiary process of thoughts about thought; Panksepp, 2009, 2010). In support of this interaction, oxytocin administration has been shown to modulate subcortical regions (e.g., amygdala, basal forebrain structures such as the bed nucleus and septal areas; Panksepp, 1992) and to alter their connectivity with cortical regions (e.g., medial prefrontal, anterior cingulate cortex, orbitofrontal cortex; Sripada et al., 2013; see Bethlehem et al., 2013, for an overview). Notably, there is evidence of widespread effects of oxytocin on the brain’s intrinsic functioning as well as of task- and context-dependency, as well as modulations by interindividual variables and sex hormones, of oxytocin’s effect on brain systems (Bethlehem et al., 2013).

Most people experience declines in certain cognitive functions as they reach advanced age, including reductions in learning efficiency, working memory, executive functioning, and cognitive processing speed. They also tend to experience greater difficulty with certain socio-affective capacities, whereas others remain largely intact (Ebner et al., 2013). For example, the ability to recognize emotions in others declines with age (Isaacowitz et al., 2007; Ruffman et al., 2008, 2012). In contrast, emotional problem solving skills (Blanchard-Fields, 2007) and some emotion-regulatory capacities (Gross et al., 1997; Scheibe and Blanchard-Fields, 2009; Shiota and Levenson, 2009; Urry and Gross, 2010) are maintained or even improve with age, and older compared to young adults report increased frequency of positive (compared to negative) feelings (Carstensen et al., 2011).

Surprisingly, though a growing literature suggests that oxytocin enhances attention to and understanding of emotions, research on oxytocin has not been linked to the literature on social and affective aging and close to nothing is known about baseline levels of oxytocin and functional changes in the oxytocin system in aging. Integrating these lines of work appears particularly fruitful, given that oxytocin’s socio-affective effects show inter-individual variations by level of proficiency (Bartz et al., 2011). That is, more impaired, compared to less impaired, individuals appear to benefit more from oxytocin administration. Relatedly, there may be a point beyond which oxytocin cannot further improve socio-affective abilities. We therefore argue that oxytocin’s effects may vary across age, as young and older adults differ in their level of socio-affective proficiency such as pertaining to emotion recognition or emotion-regulatory skills. In addition, as recently summarized (Ruffman et al., 2008; Scheibe and Carstensen, 2010; Samanez-Larkin and Carstensen, 2011; Ebner and Fischer, 2014), age-associated motivational, cognitive, and sensory changes as well as age-related structural and functional brain changes, including changes in neurotransmitter concentration and function, may influence processing of socio-affective information and underlie age variations in oxytocin’s effects (Ebner et al., 2013, 2015).

Recent studies also strongly suggest sex differences in the dynamics and actions of the oxytocin system, raising the possibility that the effects of oxytocin may be differentially regulated by gonadal steroids or other sex-specific biological factors (e.g., brain anatomy, neural processing, and/or endogenous oxytocin levels; Bos et al., 2012; Macdonald, 2013; Kanat et al., 2014; Ebner et al., 2015). Bos et al. propose a model for the neuroendocrine regulation of human social-emotional behavior according to which steroid hormones (e.g., testosterone, estrogen) and neuropeptides (e.g., oxytocin, vasopressin) interact in their effect on brain function and behavior in a dynamic variation by environmental context (e.g., perceptions of a social situation as challenging vs. safe; see also van Anders et al., 2011, for a similar construal). This mechanistic explanation is in line with a broad animal literature documenting distinct roles of oxytocin in males and females (see Macdonald, 2013, for references) as well as increasing evidence suggesting sex-opposing effects of oxytocin on amygdala reactivity (Guastella et al., 2009; Domes et al., 2010; Rupp et al., 2014), risk taking (Patel et al., 2014), emotional empathy (Hurlemann et al., 2010), cardiovascular responses to social stressors (Kubzansky et al., 2012), and kinship and competition recognition in humans (Fischer-Shofty et al., 2013).

Importantly, the majority of current research on oxytocin’s socio-affective effects has been conducted in young men, while examination of oxytocin’s socio-affective effects in women and older adults have largely been neglected (see Barraza et al., 2013; Campbell et al., 2014, for exceptions of recent studies that also comprised older adults). Thus, using a double-blind, placebo-controlled between-group design, the present study went beyond previous research by testing effects of oxytocin on meta-mood, a socio-affective capacity that shows age and sex differences, within a single experimental protocol in young and older men and women.

Meta-mood refers to the awareness about one’s capability to notice and think about one’s own feelings (attention to feelings) and the understanding of one’s own mood (clarity of feelings; Salovey et al., 1995), and can be conceptualized as a result of interrelated primary (feeling), secondary (learning and thinking), and tertiary (thoughts about thought) affect processes (Panksepp, 2010). While the majority of previous research has examined oxytocin’s effect on emotion perception in others (but see Cardoso et al., 2014), in the present study we examined its effects on subjective emotion perception in the self. We hypothesized that oxytocin’s effect on meta-mood would vary by participant age and sex. In particular, based on evidence of age and sex differences in meta-mood and emotion-regulatory skills (Gross et al., 1997; Thayer et al., 2003; Urry and Gross, 2010; Fernández-Berrocal et al., 2012), we expected beneficial effects of oxytocin on meta-mood to be most pronounced in older men, as the group characterized by the lowest level of socio-affective competence, when compared to young men and women. In contrast, we expected the effects of oxytocin to be least pronounced in older women, as the group characterized by the highest level of socio-affective competence, when compared to young women and men. We also expected oxytocin’s effects to be less pronounced in young women than young men, given that young women typically show higher socio-affective competence than young men.

## Materials and Methods

### Participants

Forty-eight young (*M* = 22.4 years, *SD* = 2.97, 18–22 years, 48% female) and 54 older (*M* = 71.2 years, *SD* = 4.92, 63–81 years, 56% female) white, English-speaking volunteers participated in the study. Participants were recruited through mailouts and fliers in the community and on campus and were screened for physical and cognitive health via self-report during an initial phone contact and an on-campus visit. Among the exclusion criteria were pregnancy, as confirmed with pregnancy testing, breastfeeding, psychological disorder, severe or progressive medical illness, known allergies to the preservatives in the nasal spray, and excessive smoking or drinking. Under the supervision of a clinical practitioner participants underwent a blood test (i.e., basic metabolic panel) and a health review covering all major bodily systems. Participants were instructed to stay well hydrated before their visit but to abstain from smoking, caffeine, alcohol, and use of recreational drugs in the 24 h, and from food, exercise, or engagement in sexual activity in the 2 h, leading up to their appointment. All test sessions took place in the mornings, typically starting around 8AM.

We recorded date of last menstruation and use of oral contraception and hormone replacement therapy. All older women were postmenopausal, all young women were premenopausal. Ten young women were in the follicular phase of their menstrual cycle. One older man and one older woman were currently on hormone replacement therapy. Seven young women were on oral contraception.

Older participants scored ≥ 30 on the Telephone Interview for Cognitive Status (Brandt et al., 1988); only 0.08% of all contacted older adults were excluded based on this criterion. Age groups were comparable in educational level. Self-reported physical functioning was good, and visual-processing speed and short-term verbal memory were comparable to typical performance levels in these age groups.

### Procedure

This data collection was part of a larger project. Participants underwent a screening phase a few days to a few weeks before the full study visit, which was partly phone-based and partly in person, and during which their overall health status was reviewed via self-report, and a blood draw was conducted. Twenty-six young (46% female) and 27 older (56% female) participants were randomly assigned to self-administer via a nasal spray 24 IUs (one puff per nostril) of oxytocin. Twenty-two young (50% female) and 27 older (56% female) participants self-administered a placebo that contained all ingredients with the exception of the oxytocin.

Numbers of young women in the follicular (oxytocin: 42%; placebo: 50%) compared to the luteal (oxytocin: 58%; placebo: 50%) phase of their menstruational cycle did not significantly differ for the two treatment groups (χ^2^ = 0.153, *p* = 0.696). Also, the two treatment groups were statistically comparable with respect to numbers of young women on oral contraceptives (oxytocin: 33%; placebo: 40%) compared to those not on oral contraceptives (oxytocin: 67%; placebo: 60%; χ^2^ = 0.105, *p* = 0.746). Furthermore, to determine comparability of the two randomly assigned treatment groups in terms of mood prior to treatment, participants indicated their positive and negative mood in the present moment via the brief Positive Affect Negative Affect Scale (PANAS; Watson et al., 1988). In particular, participants indicated on a scale ranging from 1 = *very slightly or not at all* to 5 = *extremely* for a list of adjectives (e.g., *excited, happy, afraid, alert*) how they felt at the present moment. The treatment groups reported comparable pre-treatment positive (oxytocin: *M* = 3.11, *SD* = 0.09; placebo: *M* = 3.26, *SD* = 0.11; *F*_(1,91)_ = 0.89, *p* = 0.35, ${\text{η}}_{p}^{2}$ = 0.01) and negative (oxytocin: *M* = 1.21, *SD* = 0.04; placebo: *M* = 1.19, *SD* = 0.04; *F*_(1,91)_ = 0.07, *p* = 0.80, ${\text{η}}_{p}^{2}$ = 0.00) mood.

Nasal spray administration followed recommendations for the standardized application of intranasal oxytocin (Guastella et al., 2013). Approximately 120 min after spray administration, participants responded to items of the two subscales “attention to feelings” (e.g., *I think about my mood constantly*) and “clarity of feelings” (e.g., *I am usually very clear about my feelings*) of the Trait Meta-Mood Scale (TMMS; see Salovey et al., 1995 for item inventory), on a scale ranging from 1 = *strongly disagree* to 5 = *strongly agree*. The TMMS is an introspective measure of meta-cognition associated with participants’ feelings and emotions. It allows for reliable measurement of stable attitudes and enduring qualities of the reflective experience of mood with Cronbach’s α ranging between 0.7 and 0.9 for the subscales (Salovey et al., 1995, 2002). In line with the literature (Salovey et al., 1995), the two subscales were uncorrelated (*p* > 0.05), supporting the notion that they capture largely distinct facets of meta-mood. Written informed consent was obtained following a study description. The study protocol was approved by the Institutional Review Board at University of Florida.

Examination of variable distributions indicated that one young man, one young woman, and one older man were outliers (based on 1.5 × interquartile range as defined in IBM SPSS Statistics 22) on the clarity subscale within their age (young, older) and treatment (oxytocin, placebo) group and were therefore excluded from subsequent analyses. One older woman did not fill in the questionnaire.

## Results

Mean scores in the attention and clarity subscales were examined in respective 2 (age: young, older) × 2 (sex: male, female) × 2 (treatment: oxytocin, placebo) univariate analyses of variance (ANOVAs). For significant interactions we conducted follow-up tests.

**Figure 1:**
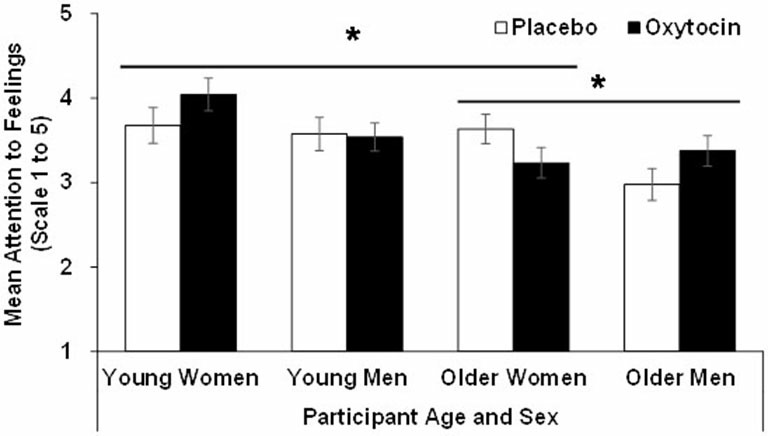
**Participant scores in the attention to feelings subscale of the TMMS in the oxytocin and placebo conditions, as a function of participant age and sex, with error bars representing mean standard errors.** *p > 0.05.

For *attention*, the effect of age (*F*_(1,90)_ = 9.23, *p* = 0.00, ${\text{η}}_{p}^{2}$ = 0.09), sex (*F*_(1,90)_ = 4.46, *p* = 0.04, ${\text{η}}_{p}^{2}$ = 0.05), and the age × sex × treatment interaction (*F*_(1,90)_ = 5.17, *p* = 0.02, ${\text{η}}_{p}^{2}$ = 0.06), were significant. That is, overall, young (*M* = 3.71, *SD* = 0.68) compared to older (*M* = 3.30, *SD* = 0.66) participants, as well as women (*M* = 3.65, *SD* = 0.71) compared to men (*M* = 3.37, *SD* = 0.65) reported more attention to feelings. However, as depicted in Figure 1, the significant three-way interaction qualified these main effects. In particular, follow-up analyses conducted separately in young and older participants showed that the sex × treatment interaction was not significant in young participants (*F*_(1,42)_ = 1.05, *p* = 0.31, ${\text{η}}_{p}^{2}$ = 0.02), while it was significant in older participants (*F*_(1,48)_ = 5.09, *p* = 0.03, ${\text{η}}_{p}^{2}$ = 0.10): in contrast to older women, oxytocin relative to placebo increased attention to feelings in older men. In addition, in women there was a significant age × treatment interaction (*F*_(1,47)_ = 4.08, *p* = 0.049, ${\text{η}}_{p}^{2}$ = 0.080), in that in young women oxytocin relative to placebo enhanced attention to feelings, while in older women oxytocin relative to placebo reduced it. There was no age × treatment interaction among men.

**Figure 2:**
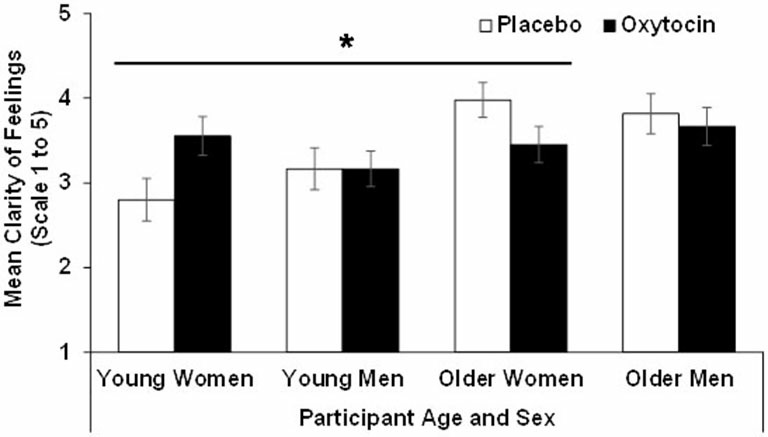
**Participant scores in the clarity of feelings subscale of the TMMS in the oxytocin and placebo conditions, as a function of participant age and sex, with error bars representing mean standard errors.** *p > 0.05.

For *clarity* the effect of age (*F*_(1,90)_ = 11.89, *p* = 0.00, ${\text{η}}_{p}^{2}$ = 0.12) and the age × treatment interaction (*F*_(1,90)_ = 4.93, *p* = 0.03, ${\text{η}}_{p}^{2}$ = 0.052) were significant, while the age × sex × treatment interaction only approached significance (*F*_(1,90)_ = 3.06, *p* = 0.08, ${\text{η}}_{p}^{2}$ = 0.03). That is, overall, older (*M* = 3.73, *SD* = 0.78) compared to young (*M* = 3.17, *SD* = 0.82) participants reported more clarity of feelings. However, the significant age × treatment interaction for clarity suggested that young participants in the oxytocin group reported more clarity of feelings than young participants in the placebo group, while the reverse held true for older participants. Similar to the findings for the attention subscale, an analysis conducted separately for men and women suggested that this age × treatment interaction only held in women (*F*_(1,47)_ = 8.08, *p* = 0.01, ${\text{η}}_{p}^{2}$ = 0.15) but not in men (*F*_(1,43)_ = 0.11, *p* = 0.74, ${\text{η}}_{p}^{2}$ = 0.00). That is, oxytocin relative to placebo enhanced clarity in young women but reduced it in older women. Results for clarity are summarized in Figure 2.

The pattern of findings remained stable when controlling, in covariate analyses (ANCOVAs), for pre-treatment mood assessed on the day of the full visit as well as processing speed, short-term verbal memory, and years of education, and use of oral contraceptive while in the study. Also, the general pattern of findings was comparable when excluding older participants on hormone replacement therapy or young women in the follicular phase, respectively.

## Discussion

There have been recent calls for systematic examination of sex and age influences on oxytocin level and function in brain and behavior (Bethlehem et al., 2013; Ebner et al., 2013, 2015; Huffmeijer et al., 2013). These calls were based on evidence that females have higher plasma oxytocin levels than males (Carter, 2007) as well as a growing literature suggesting possibly opposing effects of oxytocin on socio-affective functioning in women vs. men (see Macdonald, 2013). Also, currently, knowledge is lacking of possible differences in plasma oxtyocin levels as well as oxytocin function in human aging and preclinical evidence on aging effects on the oxytocin system is still scare and quite mixed (Fliers and Swaab, 1983; Wierda et al., 1991; Arletti et al., 1995; Arsenijevic et al., 1995; Melis et al., 1995; Parker et al., 2010). The present paper constitutes a first response to these recent calls for systematic examination of sex and age influences on oxytocin level and function (Bethlehem et al., 2013; Ebner et al., 2013, 2015; Huffmeijer et al., 2013).

To our knowledge, together with the recent study by Campbell et al. (2014), our study is the first to show an age- by sex-modulatory role of oxytocin on socio-affective functioning and thus importantly qualifies previous research that exclusively focused on young, and largely on male, participants. Results from our study offer a fruitful empirical ground on which to further explore aging effects in the oxytocin system (Ebner et al., 2013, 2015; Huffmeijer et al., 2013). In particular, our findings suggest that self-administered intranasal oxytocin exerts age- and sex-specific effects on meta-mood, that is a person’s awareness about the capability to notice and think about his/her feelings and to understand them. In contrast to older women, older men with regard to attention to feelings particularly benefitted from oxytocin treatment. This result points in the same direction as evidence from Campbell et al. (2014) who showed improved emotion recognition skills by oxytocin in older men but not older women. It is possible that oxytocin enhances communication between brain regions associated with socio-affective processing and this increased neurotransmission may be beneficial to those who are in most need (i.e., older men; Gross et al., 1997; Fernández-Berrocal et al., 2012). The sex differences in older adults are also interesting when linked to work by Rilling et al. (2014) that oxytocin treatment may render neural responses of men more similar to those of women in the placebo group. However, Rilling et al. examined effects in young adults only, while the present study showed sex difference in older adults.

In line with our Age-Related Genetic, Neurobiological, Sociobehavioral Model of Oxytocin (AGeNeS-OT) model (Ebner et al., 2013), an investigation of biological influences, such as steroids hormones or epigenetic factors related to the oxytocin receptor gene, that undergo age-related change and differ between men and women, appears particularly promising to further describe oxytocin’s differential regulation of socio-affective processes. As proposed by Bos et al. (2012), age and sex differences in neuroendocrine factors (e.g., associated with endogenous central and/or peripheral oxytocin levels, or neurotransmitters levels such as dopamine), in interaction with differences in gonadal hormones (e.g., estradiol, testosterone), influence malleability (such as via exogenous oxytocin administration) of neural connectedness between subcortical and cortical brain structures, which may underlie age-by-sex variations in oxytocin’s socio-affective effects. Also, while our understanding of oxytocin receptor expression is nascent, it is possible that age- and sex-related alterations on oxytocin receptor expression contribute to the effects observed in our study. It has been shown that estrogen upregulates oxytocin production and oxytocin receptor expressivity (Bale and Dorsa, 1997; Vasudevan et al., 2001) in the hypothalamus and other limbic brain regions. This combined with reduced release of androgens in older compared to young males may contribute to a “ramping up” effect of older men’s social and affective system after exogenous elevation of oxytocin levels via intranasal administration, and may contribute to older males’ greater attention to their own feelings in the oxytocin compared to the placebo group. Of note, the present study did not directly control for the influence of levels of gonadal hormones on the reported effects (Domes et al., 2010; Lischke et al., 2012). However, the observed pattern of findings remained after control for self-reported use of hormone replacement therapy, use of oral contraceptives, and menstrual cycle. Future studies, that collect hormone data levels in addition to neuropeptide levels and use brain imaging while men and women of different ages attend to their own feelings and process emotional information, will have to confirm possible interactive effects of hormones and neuropeptides on brain function and behavior.

Our data did not confirm previous evidence suggesting particularly greater meta-mood in older women compared to men and young women (Gross et al., 1997; Fernández-Berrocal et al., 2012). The finding, from our *post hoc* analysis, that oxytocin relative to placebo enhanced meta-mood in young women but reduced it in older women is in line with growing evidence of possible negative effects of intranasal oxytocin on socio-affective functioning under certain circumstances. For example, Cardoso et al. (2014) showed that intranasal oxytocin administration can impair emotion recognition in individuals who do not show deficits in emotion identification by promoting “oversensitivity” to emotion cues. Our findings also follow suggestions that the effects of oxytocin may be task- or context-dependent as well as modulated by interindividual differences (Panksepp, 1992; Bartz et al., 2011; Bos et al., 2012).

Young men did not exhibit a significant effect of oxytocin administration on meta-mood. While the majority of prior studies that showed beneficial effects of intranasal oxytocin in young men examined others-related cognition, such as face memory or trust perceptions, the present study focused on internally oriented meta-cognitive emotion evaluation, which may remain unaffected by oxytocin in young men. The time period between drug and questionnaire administration in our study was about 120 min, suggesting that effects of intranasal oxytocin administration may last longer than was previously assumed (Guastella et al., 2013; Paloyelis et al., 2014). However, it is possible that drug effects in the present study may not have lasted in young men resulting in null effects for this group. Future systematic investigation of age and sex differences in the timing of drug absorption and peak levels is warranted to confirm this speculation.

The current study provides first intriguing evidence of an age- by sex-neuromodulatory role of oxytocin on trait meta-mood. Neuropeptides like oxytocin only degrade slowly in the brain, which gives them the potential to have effects over longer distances involving different brain regions in a dynamic fashion (Bethlehem et al., 2013). This may imply that effects of oxytocin on more general trait meta-mood may differ from effects on more spontaneous state meta-mood. Also, previous work has suggested that the TMMS is related to more objective behavioral measures of emotional competence such as psychophysiological measures of adaptive coping (e.g., reduced cortisol release and blood pressure during acute stress; Salovey et al., 2002). Going beyond introspection as applied in the present study, it would be interesting to address in future extensions of our research effects of intranasal oxytocin on behavioral measures of emotional competence such as pertaining to emotion-regulatory capacities or emotional problem solving abilities in young and older men and women (Campbell et al., 2014).

Importantly, pre-treatment positive and negative mood was comparable in our randomly assigned treatment groups and controlling for mood did not change the pattern of findings of oxytocin’s age and sex effects on meta-mood. However, as the current study did not assess mood after oxytocin administration, it cannot rule out that oxytocin may have affected current mood, maybe in an age- and sex-differential manner, which is an interesting topic to address in future research.

In conclusion, we provide intriguing first evidence that oxytocin’s effects on meta-mood vary by age and sex. To date, close to nothing is known about the effects of intranasal oxytocin on social and emotional functions in young and older men and women (see Campbell et al., 2014, for an exception). Independent future research needs to replicate our results and determine the extent to which these modulatory effects are reflected in brain processes, such as for example associated with age and sex differences in strength of functional connectivity involved in socio-affective processing. Accumulating evidence suggests that oxytocin exemplifies one of the shared biochemical substrates that serve socio-affective functions in both humans and non-human animals and supports the notion that complex social cognition and affective functioning and its neuromodulatory control in humans can be traced back evolutionarily (Donaldson and Young, 2008; Panksepp, 2009; Pedersen et al., 2014; Ebner et al., in press). Focused cross-species comparisons in future research on oxytocin function promises great potential to unravel the principles by which the neural and genetic substrates of emotionality operate in mammalian brains, and to determine age- and sex-specific variations therein. We hope that these preliminary findings will spur future replication of oxytocin’s modulatory function in age- and sex-heterogeneous samples, with a particular focus on identification of neurobiological factors that contribute to differences in socio-affective aging and among men and women.

## Conflict of Interest Statement

The authors declare that the research was conducted in the absence of any commercial or financial relationships that could be construed as a potential conflict of interest.
